# Oral discomfort and health behavior of patients with typical vs. atypical antipsychotic drugs

**DOI:** 10.3389/fpsyt.2024.1420010

**Published:** 2024-06-26

**Authors:** Jukka H. Meurman, Heikki Murtomaa, Marja Koski

**Affiliations:** ^1^ Department of Oral and Maxillofacial Diseases, University of Helsinki, Helsinki, Finland; ^2^ Department of Oral and Maxillofacial Diseases, Helsinki University Central Hospital, Helsinki, Finland; ^3^ Department of Psychiatry, University of Helsinki, Helsinki, Finland

**Keywords:** mental health, mental disorders, antipsychotic drugs, burning mouth, oral pain, xerostomia, health behavior

## Abstract

**Introduction:**

Psychiatric patients suffer from oral diseases and side effects of antipsychotic medication. In particular, the typical antipsychotic drugs may cause severe hyposalivation with subsequent oral symptoms. We therefore aimed to compare oral health behavior and oral side effects of in-hospital patients taking typical vs. atypical antipsychotic drugs with the hypothesis that the former drugs cause more oral pain than the newer drugs.

**Methods:**

This cross-sectional questionnaire and interview study investigated subjective oral symptoms and their health behavior in 170 hospitalized psychiatric patients, comparing those taking typical vs. atypical antipsychotic drugs. Cross-tabulations and chi-square tests were used for analyses.

**Results:**

Persistent oral pain lasting throughout the day was reported by 46% in the typical, and 5% in the atypical antipsychotic group patients, respectively. In both groups, the pain was mainly in the tongue and buccal mucosa and was described as a burning sensation. A significantly higher prevalence of xerostomia was reported in the typical antipsychotic medication group (66%) compared with the atypical antipsychotic medication group (53%, p<0.01). Self-assessed dental health was assessed as poor by two-thirds of the patients of whom 69% reported toothbrushing once daily. Approximately half of them reported having had a visit to a dentist within the previous year. Of the women 28%, and of the men 17%, respectively, had received professional consultations for oral symptoms.

**Conclusion:**

The current results on psychiatrically hospitalized patients emphasize the need for awareness of oral discomfort and its subsequent effects on the quality of life in this challenging patient group. Focus should also be placed on a wide range of support encouraging the patients to maintain good daily oral hygiene and seek professional dental help when needed.

## 1 Introduction

There are limited data on oral health behavior and the effect of typical and atypical antipsychotic drugs on oral health and symptoms of the mouth even though previous studies have shown that oral health is generally poor among psychiatric patients (1, 2 3 4). Dental diseases, in particular, are prevalent in this patient group for several reasons. Daily oral self-care can be impaired and antipsychotic medication is known to cause xerostomia (2). Dry mouth is linked to various sensations in the oral mucosa such as glossodynia and burning mouth sensation (3, 4). Burning sensation specifically affects the quality of life (5).

Oral pain or burning sensation is often characterized as a burning or tingling sensation in the oral cavity in clinically normal oral mucosa (6). The pathogenesis of burning sensation of mouth mucosa is not entirely understood. However, it is thought to be due to deficiencies in both the peripheral and central nervous systems (7). Xerostomia and burning sensation often appear concomitantly but their causal relationship has not been verified (8).

Psychiatric medicines have a poor reputation regarding oral side effects due to their mechanisms of action. In particular, the anticholinergic activity of antipsychotic drugs is well known in this respect as also recently reviewed (9, 10). Derivatives of dopamine blockers are still the drugs of choice in schizophrenia and these drugs reduce salivary flow rate. Hence, many psychiatric patients indeed suffer from dry mouth due to their medication. For example, a study on elderly patients in Finland, where also this study was made, showed that the use of psychiatric drugs was the strongest explanatory factor for dry mouth, with an odds ratio (OR) of 2.1 (95% confidence interval [CI] 1.2–3.5) (11). Similarly, the same study reported that the strongest explanatory factor for burning sensation of mouth mucosa also was a psychiatric disease (OR 8.7, CI 1.4–54.1) (11).

Even though atypical antipsychotic drugs have been reported to have fewer oral side effects than typical, older antipsychotic medicines (12), only a few studies have investigated the effect of atypical antipsychotic drugs on oral discomfort. Therefore, we were interested in studying how the patients treated in a psychiatric hospital report their subjective oral symptoms depending on the nature of their medication and how were their oral hygiene practices, and subjective attitudes about oral health. Hence, the first objective was first on the two drug categories, typical and atypical antipsychotic medicines, with the hypothesis that atypical antipsychotic drugs cause fewer oral symptoms compared with typical antipsychotic drugs. Secondly, we investigated the patients´ subjective conceptions of their oral health behavior and factors related to it.

## 2 Methods

### 2.1 Patients and questionnaire

The present study included 170 patients whose treatment involved various psychiatric medications. They were patients at the Hesperia Psychiatric Hospital in Helsinki, Finland. The study data were collected using questionnaires completed by the patients in the hospital ward in the presence of a nurse. Members of the research group working in the hospital interviewed the patients and verified the medical data from them.

Of the 170 participants, 160 responded, and 136 of these responses were included in the final questionnaire data analysis. Thirty-four questionnaires (20%) were excluded due to insufficient information (e.g., the social security number and gender data were missing) or because the patient’s writing was unclear or illegible. Of the patients, 35 used both typical and atypical antipsychotic medication and were excluded from the analyses.

The structured questionnaire included 27 multiple-choice questions. A pilot study on 30 patients at the Hesperia Hospital was conducted before the current study to test and subsequently revise the questionnaire. The World Health Organization International Classification of Diseases (ICD-10) and the Finnish catalog of drugs (“Pharmaca Fennica”) were used to categorize the patients’ diseases and medications, respectively. An informed consent form was signed by all patients in the study, and the ethical principles of the Declaration of Helsinki were adhered to throughout the investigation. Ethical approval was attained from the internal review board of the University of Helsinki as well as from hospital management of the Hesperia Psychiatric Hospital in Helsinki (Ethical Permit No. 15/1999, 266§).

### 2.2 Statistical methods

The data were analyzed using SPSS for Windows 11.01 (Chicago, IL, USA). The patients’ reported symptoms were compared with their underlying illnesses and medications. The type of psychiatric medication (typical vs. atypical antipsychotic drug therapy), sex, and age were analyzed using cross-tabulations and chi-square tests. The level of significance was set at p<0.05.

## 3 Results

### 3.1 Basic characteristics of the patients and the use of medication

According to the employment history, nearly half of the patients were on pension (43%) and one-fifth were unemployed with no differences in the background variables. No vocational training was reported by 22% and at least a college-level education by 30% of the respondents.

**Figure 1:**
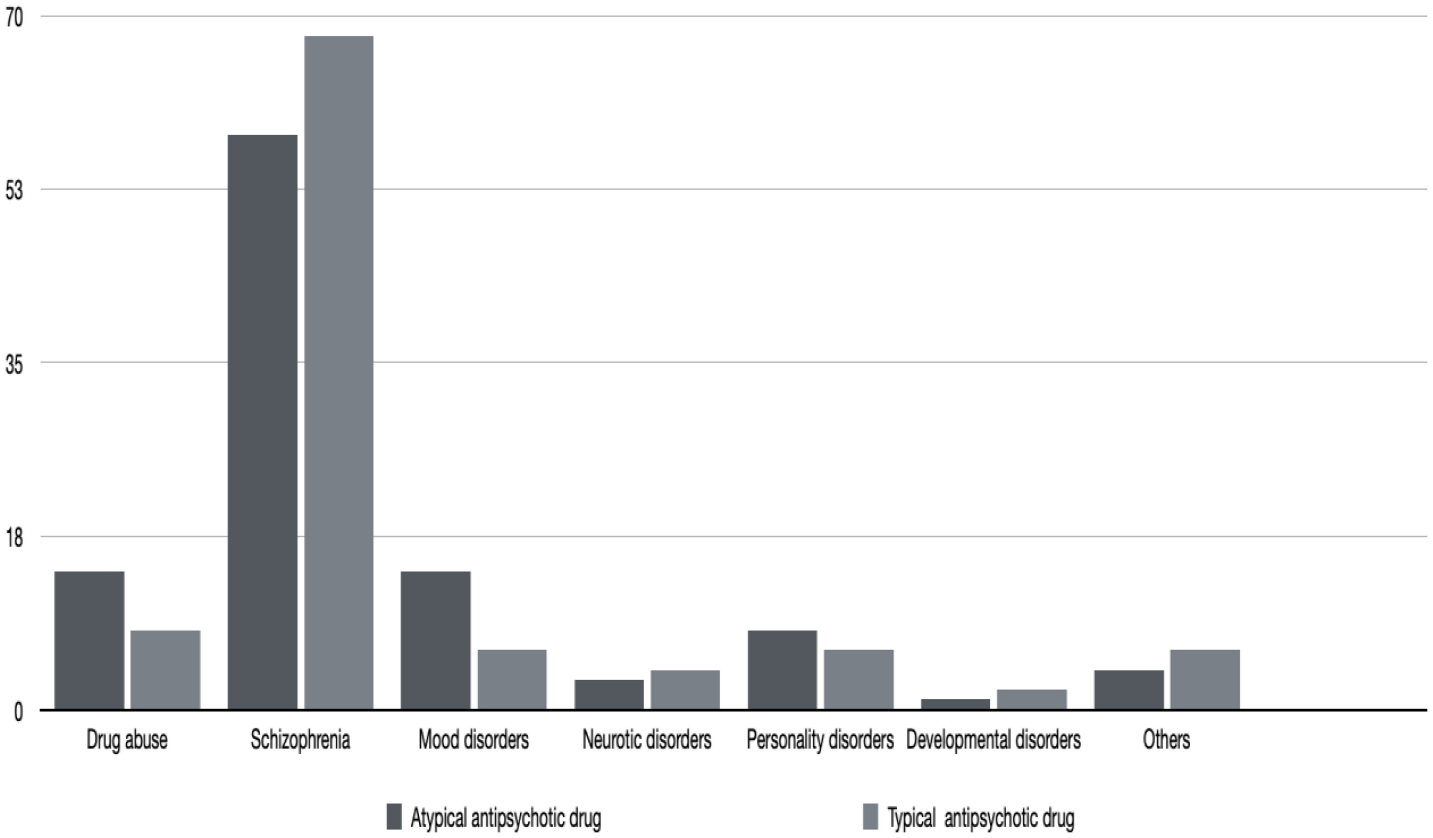
Diagnoses between the typical and atypical antipsychotic medication groups.

On average, patients taking atypical antipsychotic medication were younger than those taking typical antipsychotic drugs, but the difference was not statistically significant. **Table 1** and **Figure 1** give the details of the patients in the two groups. None of the patients reported being edentulous. Schizophrenia and mood disorders were the most common diagnoses in patients taking typical antipsychotic drugs, while schizophrenia alone was the most common diagnosis among the patients taking atypical antipsychotic medication. **Table 2** gives the drug categories taken by the patients. A commonly used typical antipsychotic drug was levomepromazine, while in the atypical antipsychotic drug group, it was risperidone.

**Table 1:** Basic characteristics of the patients.

	Typical antipsychotic drug group (n = 94)	Atypical antipsychotic drug group (n = 66)	Significance
**Mean age with SD (years)**	40.7 ± 11.4	37.0 ± 11.2	ns.
>40-year-old	52%	35%	p = 0.001
**Women (n, %)**	40 (42%)	38 (58%)	p < 0.01
**Main psychiatric diagnoses**			p = 0.041
Schizophrenia	77.7%	87.9%	
Mood disorder	89.5%	9.1%	
Drug or alcohol abuse	18.1%	10.6%	
**Main medications**			p = 0.006
Schizophrenia	79.3%	89.2%	
Mood disorder	18.5%	9.2%	
Drug or alcohol abuse	18.5%	10.8%	

**Table 2:** The distribution of most used drugs (in percent).

	Women	Men	All
Typical antipsychotic drug group			
Levomepromazine	28,8%	22,0%	25,4%
Chlorpromazine	11,9%	25,4%	18,6%
Perphenazine	8,5%	11,9%	10,2%
Haloperidol	8,5%	3,4%	5,9%
Atypical antipsychotic drug group			
Risperidone	27,1%	13,6%	20,3%
Clozapine	16,9%	18,6%	17,8%
Olanzapine	15,3%	11,9%	13,6%

### 3.2 Reported oral symptoms

**Table 3** gives the reported symptoms, their location, and the character of the pain. There was no difference in the persistent, all-day-long oral pain between the groups. Patients taking typical antipsychotic drugs reported slightly more often severe pain than those taking atypical antipsychotic medication. The location of oral pain was the same in both groups. It needs to be re-emphasized that none of the patients reported having lost all their teeth. There were no data about eventual dentures.

**Table 3:** Reported symptoms of the mouth (in percent).

	Typical antipsychotic drug group	Atypical antipsychotic drug group	Significance
**All day long pain in mouth**	45%	43%	
Mild	53%	60%	
Moderate/severe	47%	40%	
**Xerostomia** (%)	66%	53%**	p < 0.01
In the morning	30%	21%	
All day long	36%	13%	
Eating problems due to dry mouth	41%	32%	
**Pain in mouth**			p = 0.996
By waking up	24%	24%	
Later in the morning	31%	33%	
All day long	45%	38%	
**Location of pain**			p = 0.741
Border of tongue	19%	19%	
Surface of tongue	14%	19%	
Tip of tongue	12%	7%	
Bottom of mouth	14%	7%	
Palate	12%	16%	
Buccal mucosa	19%	19%	
Gingival	9%	13%	
Not specified	2%	0%	

** significant difference.

The prevalence of xerostomia is also given in **Table 3**, showing that 66% of the patients in the typical antipsychotic drug group and, respectively, 53% of the patients in the atypical antipsychotic drug group suffered from dry mouth. Despite the high prevalence of xerostomia in both patient groups, eating problems were infrequently reported. 41% in the typical antipsychotic drug group and 38% in the atypical antipsychotic drug group, respectively, reported difficulties in eating because of oral symptoms.

Persistent xerostomia was more common in the group taking typical antipsychotic medication (36%) compared with the atypical antipsychotic drug group (13%) and it was more common in women of both medication groups. Dry mouth was more severe in patients taking typical antipsychotic drugs and medications for mania; it was also prevalent in patients who took several drugs daily. However, these differences were not statistically significant (results not shown).

All patients drank water to relieve their dry mouth symptoms, but artificial saliva preparations were also frequently used. Other remedies such as lozenges, pastilles, olive oil, or oral gels were infrequently used. Of the women, 26% and of the men 13%, respectively, had received professional consultations for dry mouth. However, measuring salivary secretion rates and analyzing saliva, in general, had been conducted only for nine women and five men.

### 3.3 Health behavior and oral health concepts

Regular smoking was reported by 53% of women and 73% of men with no significance to reported oral health behavior. Slightly over half of the patients reported smoking 11- 20 cigarettes daily. None of the patients reported having lost all of their teeth and none had not lost any of their teeth. The self-assessed dental health was reported to be good by 35% of the patients. The respective figures for average and poor dental health were 24% and 35%. In practice, all the patients used toothbrushes to clean their teeth although interdental cleaning was rare (**Table 4** **).** Toothbrushing at least once daily was reported by 69%; women were more active in more than once a day brushing their teeth (61% (p <0.05) and in regular use of toothpaste (84%) (p <0.01) than men (25% and 59%, respectively). Approximately half of the patients reported having had a visit to a dentist within the previous year with no statistically significant differences in the background. The vast majority (80%) had contacted the dentist on their initiative. The mode of treatment during the latest visit was examination (58%), restorations (56%), and preventive/periodontal therapy (24%).

**Table 4:** Self-reported oral health behavior of the 136 patients (in percent).

	Sex		Age		Patients
	Women	Men	<40 yrs.	>40 yrs.	Total 136
Teeth cleaning products used					
Toothbrush	97	96	96	97	96
Toothpicks	33	30	25	38	32
Dental floss	24	15	24	15	20
Toothbrushing frequency					
Never	0	6	2	5	3
Less often than every other day	14	24	13	24	19
Every other day	9	9	8	11	9
Once per day	17	36	30	23	26
More often than once per day	61	25*	48	38	43
					**100**
Toothpaste use					
Regularly	84	59*	72	70	71
Often	13	21	15	19	17
Occasionally	3	15	10	8	9
Never	0	6	3	3	3
					**100**

*Statistically significant difference between genders p>0.01.

## 4 Discussion

Patients with serious mental illness need a wide range of support with a multidisciplinary approach to maintain their oral health which has a strong impact on quality of life, daily functioning, and social behavior (13, 14). Performing manual oral hygiene practices may be challenging and the patients may feel ashamed of their inability to take care of their oral health (15). Furthermore, antipsychotic medicines with their anticholinergic activity causing xerostomia increase the risk of developing oral diseases and characteristic symptoms (9).

Investigating the subjective oral symptoms of hospitalized psychiatric patients according to the type of antipsychotic medication they were taking was the main objective of this descriptive study. Xerostomia indeed was more prevalent in patients taking typical antipsychotic drugs compared with patients taking atypical drugs. This finding confirms our study hypothesis. The result could reflect the different receptor affinities of the two drug categories, which is a highly complex issue and beyond the scope of the present article (16). It should be mentioned that paradoxical hypersalivation has also been associated with the use of antipsychotic drugs but this was not reported by our patients (17). Furthermore, it was interesting to note that the atypical antipsychotic medication had mainly been prescribed to younger patients whose disease history was anticipated to be shorter than that of older patients, who likely had been taking typical antipsychotic drugs for several years.

Pain in the mouth was reported by patients of both the groups at about the same frequency although all-day long pain was more often reported by those taking typical antipsychotic medication. This was unexpected when considering the analgesic effect of typical antipsychotic drugs in general (18). Dry mouth and burning sensation of mouth mucosa often occur concomitantly, and stimulation therapy for salivary flow has been shown to relieve the burning symptoms, too (19). Xerostomia and oral pain were more prominent problems among the psychiatric patients in the present study compared with other groups of severely ill patients like those with cancer (20), or patients with liver diseases (21). A Finnish national health survey of subjects 30 years of age or older reported that burning sensation and eating problems (dysphagia) are associated with the number of daily medications taken by patients (22).

The effects of typical antipsychotic medications are mediated by at least four mechanisms. Typical antipsychotics inhibit dopamine-2 receptors, muscarinic cholinergic receptors, alpha-adrenergic receptors, and histamine receptors. In particular, medications that block muscarinic receptors induce oral dryness (23). Numerous psychopharmacological investigations have been conducted on antipsychotic drugs such as clozapine, risperidone, olanzapine, sertindole, and quetiapine, including studies examining the binding of neuroleptics to dopamine, serotonin, and muscarinic receptors, as well as studies on novel treatment modalities (24). A recent Lancet Editorial emphasized the need for developing new drugs for the treatment of schizophrenia but until such medications become available one must live with the oral side effects of current antipsychotic drugs (25).

It should be further discussed, that conducting a study among hospitalized patients with severe psychiatric diseases is challenging due to several obstacles. These include problems with patient management and the often-limited capacity of these patients to understand the study protocol. Furthermore, participants in questionnaire studies tend to provide favorable responses, a phenomenon referred to as social desirability answering, which may affect the respondents’ answers (26). However, the reported behaviors and conceptions should be interpreted rather as overestimations than underestimations. Additionally, 80% of the collected questionnaires were available for our analyses; this rate is satisfactory for this type of investigation. A definite weakness of our study was the fact that we did not have oral/dental clinical status of the patients. Factors like having dentures, for example, might have been interesting to know because these can affect the sensations of the mouth. In the retrospect, this question should have been in the questionnaire used.

There is sufficient scientific evidence that oral diseases are risk indicators for systemic diseases sharing the same risk factors with other major non-communicable diseases (27). Thus it was interesting to note that only one-third of the patients assessed their oral health as good. Less than half did not reach the general recommendation of twice-daily toothbrushing. Nevertheless, many patients had received professional consultations for their oral symptoms and health problems, but very few had had actual saliva tests performed. Measuring salivary flow rate among xerostomia patients, to get an objective status, is considered necessary in modern dentistry (28). In general, dental health among psychiatric patients indeed is poor, and their ability to seek and obtain proper dental treatment must be addressed (29–34). Also in this regard, the issue of patient quality of life cannot be overemphasized (35).

## 5 Conclusion

This study showed that patients with typical antipsychotic medication reported more oral side effects than those using atypical antipsychotic medication. Oral pain and xerostomia were the characteristic symptoms in both medication groups. Xerostomia was particularly prevalent among the patients taking typical antipsychotic medication. Many patients also assessed their oral health as poor. In the treatment choice of antipsychotic medication, the high prevalence of oral side effects and their effect on the patient´s quality of life should be taken into account. Emphasis should also be put on encouraging the patients to take good care of their oral hygiene daily and seek dental help when needed.

## Data Availability

The datasets presented in this article are not readily available due to the sensitive patient material and ethical restrictions. Requests to access the datasets should be directed to jukka.meurman@helsinki.fi.

## In memoriam

This article is based on a questionnaire and interview investigation of psychiatric inpatients of the Hesperia Hospital, Helsinki, Finland. The study was conducted by the late psychiatrist Heikki Rantanen, with dentist Kaija Koski, who both passed away after completing the interviews before the COVID-19 epidemic. The current authors, who supervised the study, gratefully acknowledge their work, now in memoriam. We then analyzed the results and put the work in writing.
