# Correction: Oral discomfort and health behavior of patients with typical vs. atypical antipsychotic drugs

**DOI:** 10.3389/fpsyt.2026.1904298

**Published:** 2026-07-09

**Authors:** Jukka H. Meurman, Heikki Murtomaa, Marja Koski

**Affiliations:** 1 Department of Oral and Maxillofacial Diseases, University of Helsinki, Helsinki, Finland; 2 Department of Oral and Maxillofacial Diseases, Helsinki University Central Hospital, Helsinki, Finland; 3 Department of Psychiatry, University of Helsinki, Helsinki, Finland

**Keywords:** mental health, mental disorders, antipsychotic drugs, burning mouth, oral pain, xerostomia, health behavior

Author “Marja Koski” was omitted as an author in the published article. This was because she was erroneously thought to have passed away and could not be reached by the time of writing the article. Therefore, she was only mentioned in the In Memoriam paragraph of the published article. She is thankfully well and has now been contacted. She agrees with her name to be put in the Correction.

The correct **Author list** for this article reads:

“Jukka H. Meurman^1,2^ Heikki Murtomaa^1^ and Marja Koski^3^

1Department of Oral and Maxillofacial Diseases, University of Helsinki,

2Helsinki University Central Hospital, Helsinki, Finland,

^3^Department of Psychiatry, University of Helsinki, Helsinki, Finland”

The article’s **Citation** has been updated to read:

“Meurman JH, Murtomaa H and Koski M (2024) Oral discomfort and health behavior of patients with typical vs. atypical antipsychotic drugs. *Front. Psychiatry* 15:1420010. doi: **10.3389/fpsyt.2024.1420010**”

The article’s **Copyright statement** has been updated to read:

“© 2024 Meurman, Murtomaa and Koski. This is an open-access article distributed under the terms of the **Creative Commons Attribution License (CC BY)**. The use, distribution or reproduction in other forums is permitted, provided the original author(s) and the copyright owner(s) are credited and that the original publication in this journal is cited, in accordance with accepted academic practice. No use, distribution or reproduction is permitted which does not comply with these terms.”

The **In Memoriam** statement has been corrected to read:

“This article is based on a questionnaire and interview investigation of psychiatric inpatients of the Hesperia Hospital, Helsinki, Finland. The study was conducted by the late psychiatrist Heikki Rantanen, with dentist Kaija Koski, who both passed away after completing the interviews before the COVID-19 epidemic. The current authors, who supervised the study, gratefully acknowledge their work, now in memoriam. We then analyzed the results and put the work in writing.”

The **Author Contributions** statement has been corrected to add contributions for Marja Koski:

“MK: Investigation, Methodology, Writing – original draft”

The original version of this article has been updated.

